# Supplementary material for: Roads affect the spatial structure of butterfly communities in grassland patches
Source: PeerJ. 2018 Aug 14;6:e5413. doi: 10.7717/peerj.5413 (PMC6097499; doi:10.7717/peerj.5413)
Supplement: Supplemental Information 1 — Additional results for butterfly and plants. [file peerj-06-5413-s001.docx]

**Supplementary information 1**

**
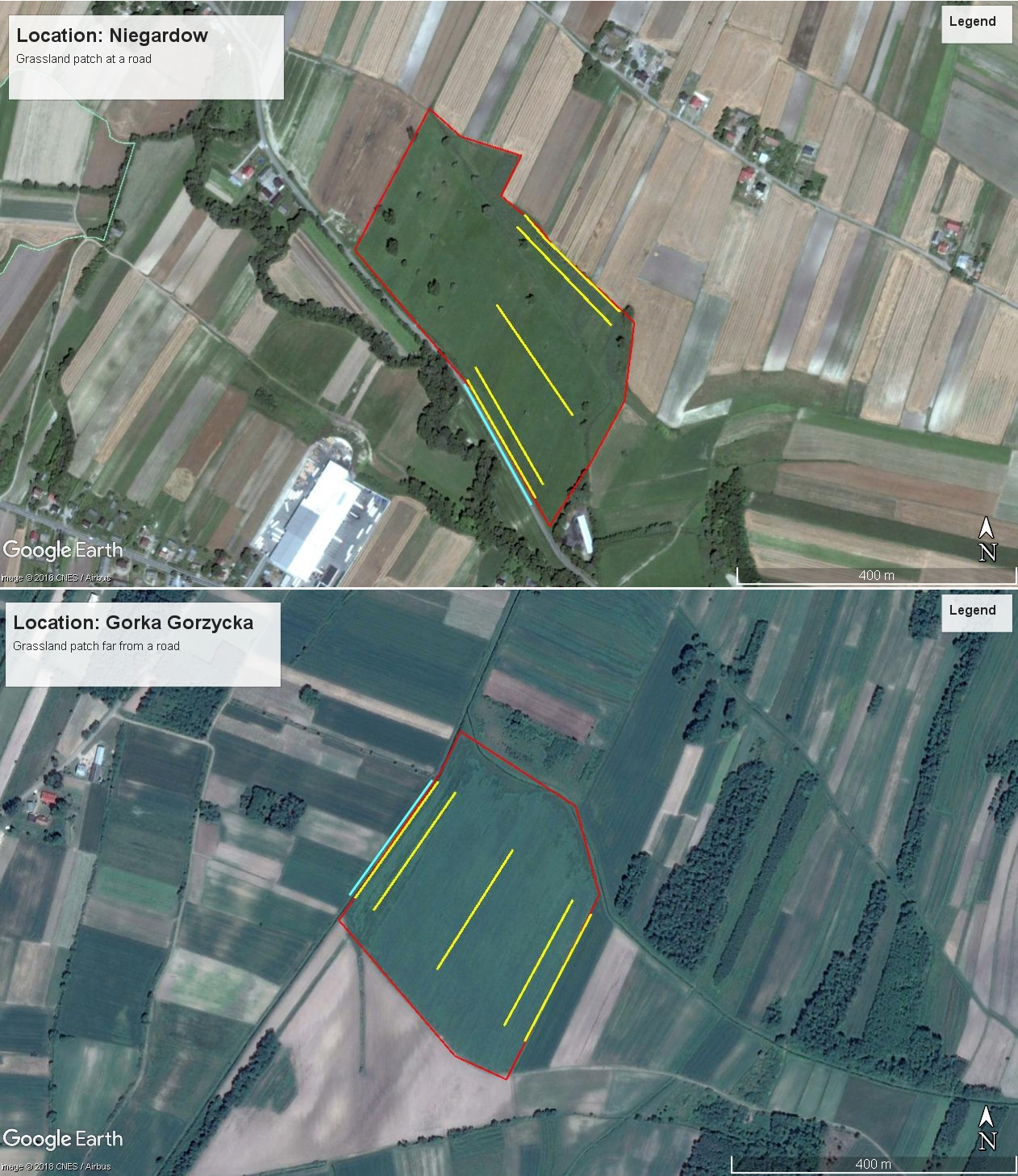
**

**Figure S1.** Photos (Google Corp. 2018) of exemplary studied grassland patches. Upper panel: a grassland patch adjacent to a road with traffic in Niegardów, Southern Poland. Lower panel: a reference grassland patch located far from paved road located in Górka Gorzycka, Southern Poland. Red lines indicate grassland patch boundaries, yellow lines indicate location of transects where live butterflies were counted, bluish line indicate a transect where dead butterflies were collected on a road.


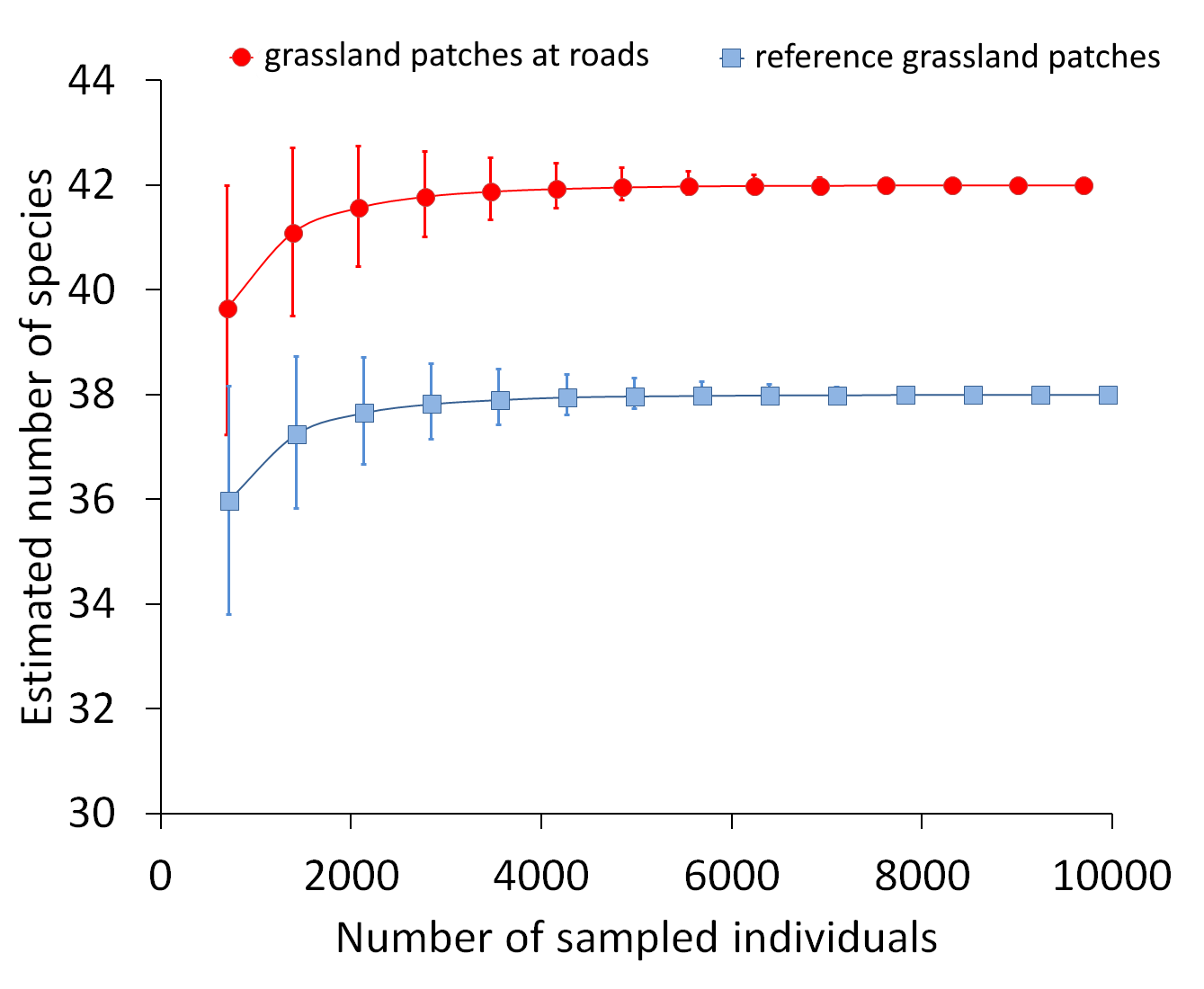


**Figure S2.** Rarefaction depicting the estimated number of species as the function of number of sampled individuals in grassland patches adjacent to roads (red) and reference grassland patches located far from roads (blue). Whiskers are 95% confidence intervals.


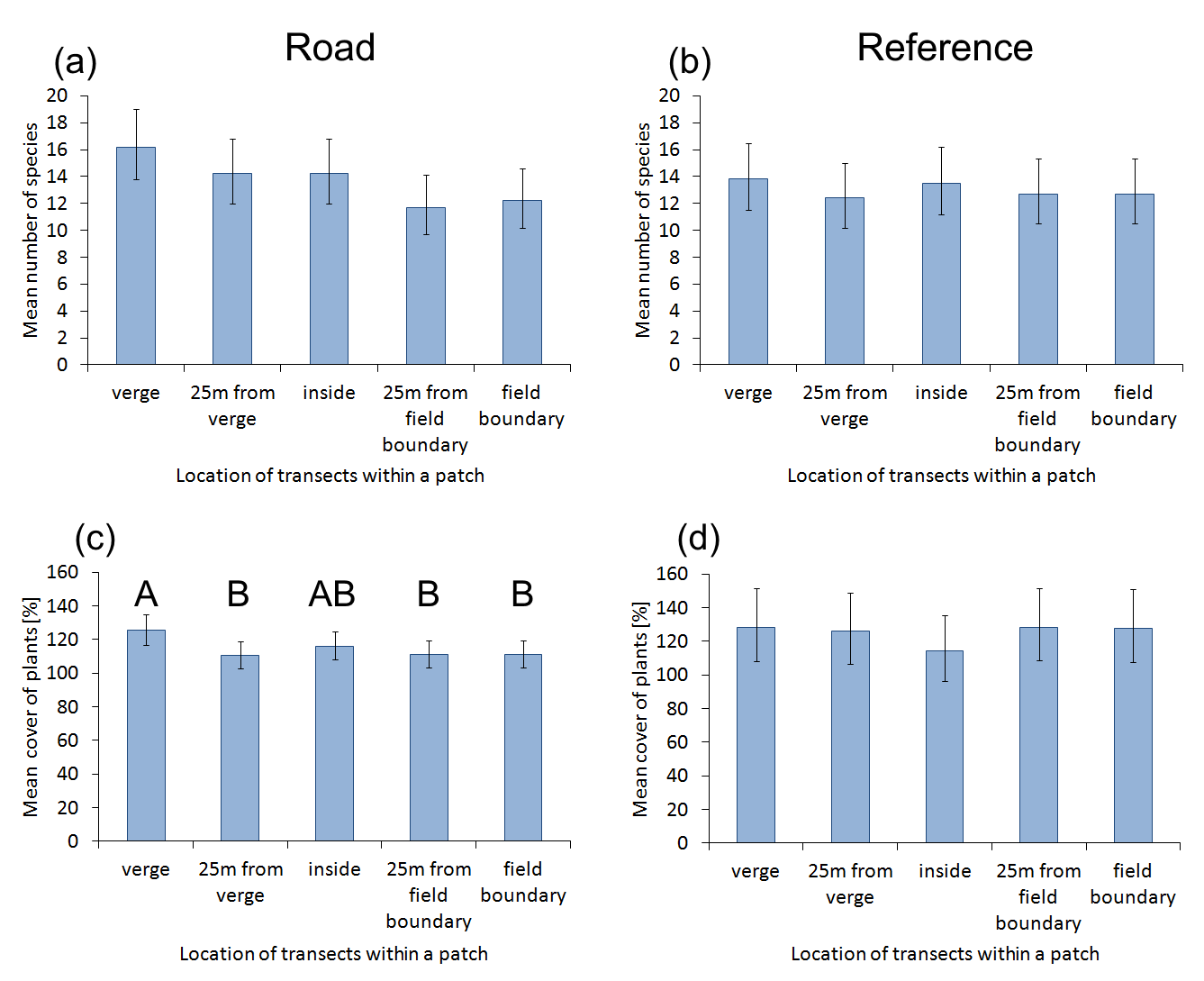


**Figure S3.** The impact of transect location on mean number of plant species (a,b) and their cover (c,d) within grassland patches bordering with road (a,c) and apart from roads (b, d). Whiskers are 95% confidence intervals. The only statistically significant difference were found for plant cover in grasslands at roads (c): levels not connected by the same capital letter denote statistically significant differences.


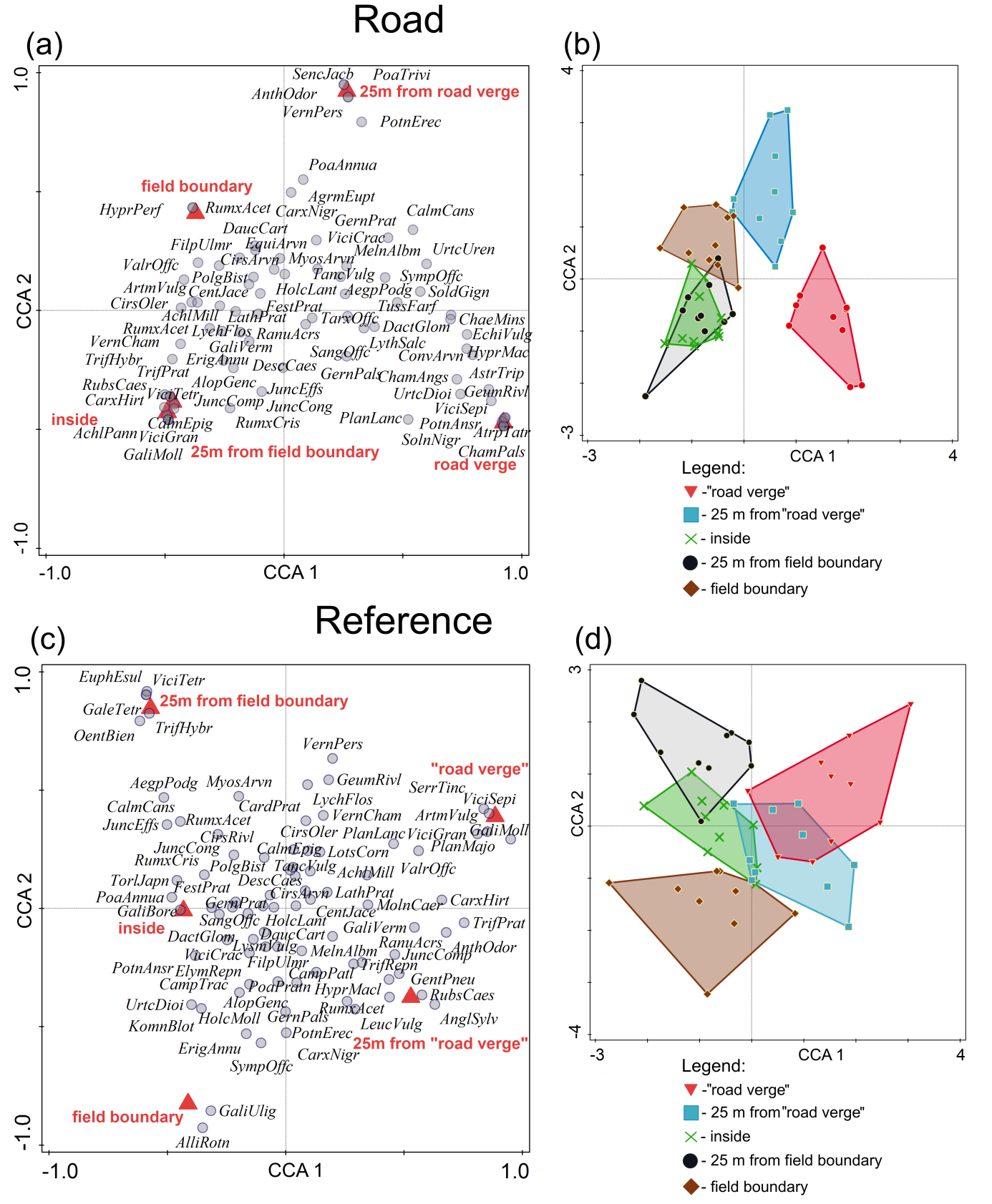


**Figure S4.** Ordination of plant species in the canonical ordination analysis in different parts of the grassland patches bordering with roads (a, b) and apart from a road (c, d). Explanation: road verge – transect on a road verge, 25 m from road verge - transect located inside the grassland patch 25 m from a road verge, inside – transect located in the interior of a grassland patch, 25 m from field boundary – transect located inside grassland patch 25 m from a border between the patch and arable field, field boundary – transect located at the border between the habitat patch and arable field. In case of reference grassland patches (grasslands located far from a road) “road verge” was a transect located along a field road used by farmers. Species abbreviations are first letters of the genus and species names.

**
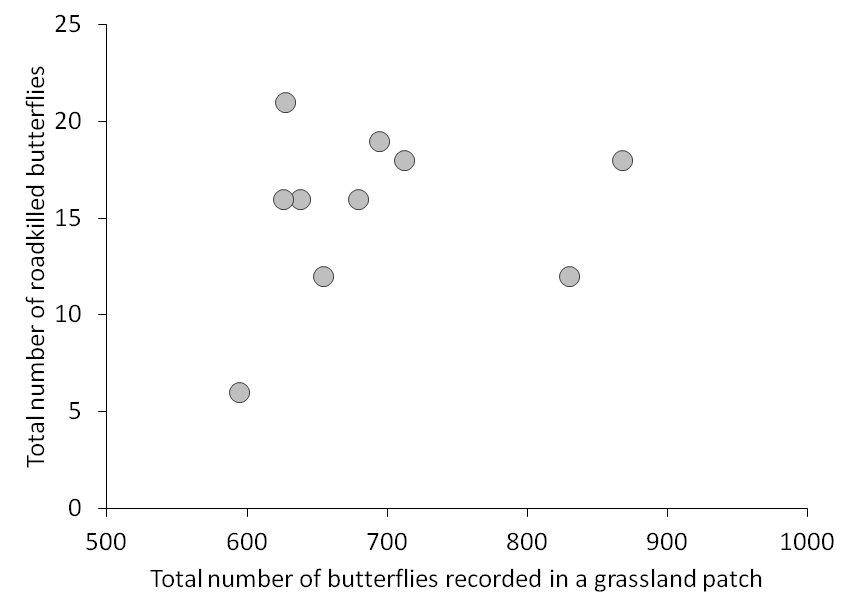
**

**Figure S5.** Relationship between the total number of living butterflies recorded in grassland patches located at roads and number of roadkilled butterflies. Correlation coefficient was statistically non-significant (see main text).


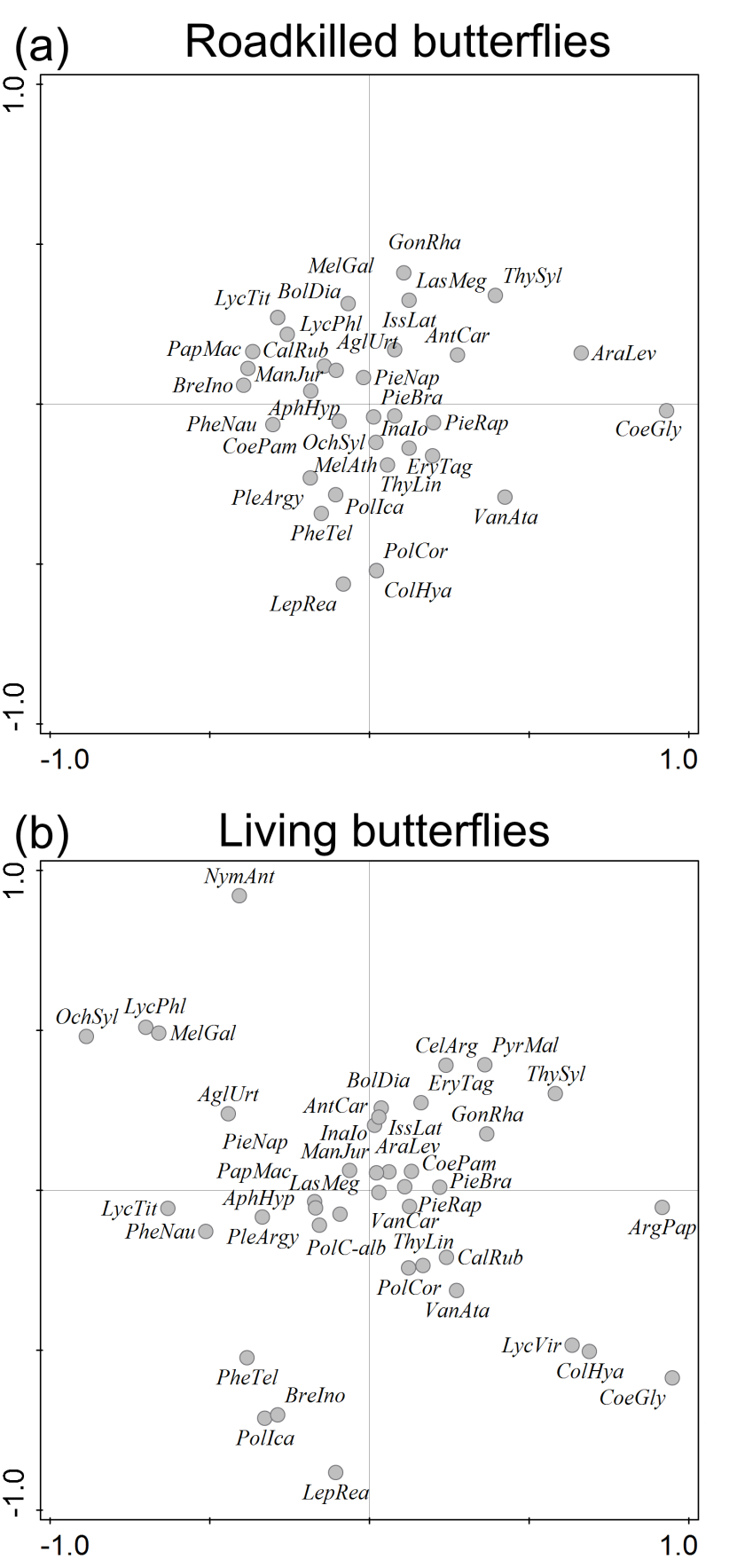


**Figure S6.** Co-correspondence ordination of roadkilled butterflies (a) via community of living butterflies on road verges (b) in grassland patches located at roads

**Table S1.** The list of all butterfly species and their abundance in grassland patches adjacent to roads and grassland patches located far from roads. Species are sorted in a decreasing order according the total abundance

| Species | Road | Reference | Total | % |
| --- | --- | --- | --- | --- |
| *Pieris rapae* | 728 | 907 | **1635** | **11.7** |
| *Coenonympha pamphilus* | 765 | 707 | **1472** | **10.5** |
| *Aphantopus hyperantus* | 451 | 512 | **963** | **6.9** |
| *Gonepteryx rhamni* | 257 | 692 | 949 | **6.8** |
| *Polyommatus icarus* | 406 | 463 | **869** | **6.2** |
| *Inachis io* | 190 | 556 | **746** | **5.3** |
| *Anthocaris cardamines* | 342 | 304 | **646** | 4.6 |
| *Maniola jurtyna* | 335 | 282 | 617 | 4.4 |
| *Pieris brassicae* | 271 | 218 | 489 | 3.5 |
| *Pieris napi* | 328 | 156 | 484 | 3.5 |
| *Thymelicus lineola* | 235 | 212 | 447 | 3.2 |
| *Aglais urticae* | 253 | 130 | 383 | 2.7 |
| *Erynnis tages* | 175 | 175 | 350 | 2.5 |
| *Papilio machaon* | 187 | 140 | 327 | 2.3 |
| *Melanargia galathea* | 138 | 189 | 327 | 2.3 |
| *Araschnia levana* | 153 | 170 | 323 | 2.3 |
| *Vanessa atalanta* | 150 | 89 | 239 | 1.7 |
| *Issoria latonia* | 165 | 74 | 239 | 1.7 |
| *Pyrgus malvae* | 102 | 112 | 214 | 1.5 |
| *Lycaena phlaeas* | 106 | 105 | 211 | 1.5 |
| *Thymelicus sylvestris* | 144 | 67 | 211 | 1.5 |
| *Boloria dia* | 116 | 89 | 205 | 1.5 |
| *Brenthis ino* | 56 | 144 | 200 | 1.4 |
| *Lassiomata megera* | 125 | 51 | 176 | 1.3 |
| *Vanessa cardui* | 79 | 80 | 159 | 1.1 |
| *Lycaena tityrus* | 90 | 68 | 158 | 1.1 |
| *Phengaris nausithous* | 82 | 67 | 149 | 1.1 |
| *Phengaris teleius* | 60 | 85 | 145 | 1.0 |
| *Ochlodes sylvanus* | 55 | 55 | 110 | 0.8 |
| *Colias hyale* | 68 | 40 | 108 | 0.8 |
| *Coenonympha glycerion* | 52 | 52 | 104 | 0.7 |
| *Leptidea reali* | 50 | 31 | 81 | 0.6 |
| *Callophrys rubi* | 57 | 0 | 57 | 0.4 |
| *Polyommatus coridon* | 34 | 10 | 44 | 0.3 |
| *Lycaena virgaureae* | 27 | 16 | 43 | 0.3 |
| *Celastrina argiolus* | 15 | 15 | 30 | 0.2 |
| *Argynnis paphia* | 17 | 10 | 27 | 0.2 |
| *Polygonia c-album* | 8 | 17 | 25 | 0.2 |
| *Melithea athalia* | 17 | 5 | 22 | 0.2 |
| *Plebeius argyrognomon* | 20 | 0 | 20 | 0.1 |
| *Nymphalis antiopa* | 8 | 0 | 8 | 0.1 |
| *Argynnis aglaja* | 5 | 0 | 5 | 0.0 |
| Total | 6922 | 7095 | 14017 | 100.0 |
